# Influence of the amino-terminal sequence on the structure and function of HIV integrase

**DOI:** 10.1186/s12977-020-00537-x

**Published:** 2020-08-31

**Authors:** Grant Eilers, Kushol Gupta, Audrey Allen, Jeffrey Zhou, Young Hwang, Michael B. Cory, Frederic D. Bushman, Gregory Van Duyne

**Affiliations:** 1 Department of Microbiology, Perelman School of Medicine, University of Pennsylvania, Philadelphia, PA USA; 2 Department of Biochemistry and Biophysics, Perelman School of Medicine, University of Pennsylvania, Philadelphia, PA USA

**Keywords:** Integrases, Retroviridae, HIV, X-ray crystallography, Protein structure, Biophysics

## Abstract

**Background:**

Antiretroviral therapy (ART) can mitigate the morbidity and mortality caused by the human immunodeficiency virus (HIV). Successful development of ART can be accelerated by accurate structural and biochemical data on targets and their responses to inhibitors. One important ART target, HIV integrase (IN), has historically been studied in vitro in a modified form adapted to bacterial overexpression, with a methionine or a longer fusion protein sequence at the N-terminus. In contrast, IN present in viral particles is produced by proteolytic cleavage of the Pol polyprotein, which leaves a phenylalanine at the N-terminus (IN 1F). Inspection of available structures suggested that added residues on the N-terminus might disrupt proper protein folding and formation of multimeric complexes.

**Results:**

We purified HIV-1 IN 1F^1–212^ and solved its structure at 2.4 Å resolution, which showed extension of an N-terminal helix compared to the published structure of IN^1–212^. Full-length IN 1F showed increased in vitro catalytic activity in assays of coupled joining of the two viral DNA ends compared to two IN variants containing additional N-terminal residues. IN 1F was also altered in its sensitivity to inhibitors, showing decreased sensitivity to the strand-transfer inhibitor raltegravir and increased sensitivity to allosteric integrase inhibitors. In solution, IN 1F exists as monomers and dimers, in contrast to other IN preparations which exist as higher-order oligomers.

**Conclusions:**

The structural, biochemical, and biophysical characterization of IN 1F reveals the conformation of the native HIV-1 IN N-terminus and accompanying unique biochemical and biophysical properties. IN 1F thus represents an improved reagent for use in integration reactions in vitro and the development of antiretroviral agents.

## Background

Integration of a reverse-transcribed DNA copy of the HIV RNA genome into a host cell chromosome is an essential step in retroviral replication [1]. The integrated provirus serves as a template for retroviral gene expression and the production of a new generation of virions. Integration also establishes the potential for latency, a major barrier to the treatment and cure of HIV-1 infection. Integrase (IN), the retroviral enzyme that catalyzes integration, is produced by proteolysis of the viral Gag-Pol polyprotein precursor by the virus-encoded protease. HIV-1 IN is comprised of three domains: the N-terminal (NTD), catalytic core (CCD), and C-terminal (CTD) domains. Some non-lentiviral INs contain an additional N-terminal extension domain [2]. The NTD adopts a zinc finger fold containing a conserved HHCC motif essential for activity [3–5]. The CCD contains a D,D-35-E motif which binds divalent metal ions within an RNase H-like fold that comprises the active site [6–8]. The CTD adopts an SH3-like fold and is implicated in DNA binding [9, 10]. Dimers of each isolated domain have been observed [3, 8, 10–12] and recombinant full-length IN has been reported to exist in forms ranging from monomer to octamer [13–18].

IN carries out two catalytic reactions: 3′-processing and strand transfer [1], in a macromolecular complex consisting of multiple IN protomers, viral DNA, cofactors, and host cell proteins termed the intasome [14, 19–25]. Early intasome structures were determined with IN from the prototype foamy virus (PFV) [26–30], revealing the structural details of enzymatic activity and the mechanism of action of the strand-transfer inhibitors (STIs), which displace the 3′ viral DNA end from the active site, rendering the intasome nonfunctional [22, 23, 31, 32]. STIs are in widespread clinical use [33], however, as with all antiretrovirals, development of resistance is a major barrier to durable inhibition of viral replication [34, 35].

PFV intasome structures and homology modeling [36, 37] have provided important insight into HIV-1 intasome function; however, PFV IN diverges significantly from HIV-1 IN in sequence identity, interdomain linker length, and the presence of an N-terminal extension domain [38]. Intasome structures from Rous sarcoma virus (RSV) [39], mouse mammary tumor virus (MMTV) [40], and Maedi-visna virus (MVV) [25] have furthered our understanding of HIV-1 IN. In contrast to PFV IN [26, 41], study of HIV-1 IN is challenging due to poor solubility and a propensity to aggregate [42, 43], along with inefficient catalysis in vitro [44–50]. Fusion of a small DNA-binding protein, Sso7d, that mimics the N-terminal extension domain of PFV to the N-terminus of HIV-1 IN improved catalysis and the solubility of HIV-1 intasomes [51], and enabled structural determination of the HIV-1 core intasome complex by cryo-electron microscopy (cryo-EM) [23, 24]. However, a native HIV-1 intasome structure remains elusive.

The first N-terminal residue of HIV-1 IN is a highly conserved phenylalanine [52–54] liberated by retroviral protease cleavage from the C-terminus of reverse transcriptase. Viruses containing engineered substitutions at IN F1 are replication-incompetent [55], showing defects in reverse transcription and integration, characteristic of class II IN mutations such as those that disrupt the HHCC motif [56–59]. Another closely studied NTD substitution Y15A also affects reverse transcription and integration [60], and IN^Y15A^ is hypo-oligomeric in solution [13, 61]. Isolated IN NTD^Y15A^ is structurally constrained, adopting only one of two NTD conformational states (the E form) [62] while the wild type NTD adopts both the E and D forms [3]. Conformational transition between E and D forms involves significant structural rearrangements in the NTD, including a change in the length of the ɑ1 helix by 6 residues [3]. The aberrant phenotypes caused by substitutions at F1 and Y15 led us to investigate the structure and function of the HIV-1 NTD in more detail.

IN is often produced for laboratory studies by bacterial overexpression in vitro with an N-terminal methionine (IN MF) [61, 63, 64] or as an N-terminal fusion protein, such as the Sso7d-IN fusion [23, 24, 51]. Solution structures of the isolated NTD were determined from constructs purified with a cleavable N-terminal affinity tag [3, 65], so that thrombin cleavage of the fusion protein left three residues (G-S-H-) preceding F1 (IN GSH). In the solution structure of IN GSH_NTD_ [3], the backbone carbonyl of F1 contributes the first hydrogen bond of the ɑ1 helix. The solution structure of another variant, IN GSH_NTD_^H12C^, which contains a substitution in the HHCC Zn-binding motif, shows a different N-terminal structure: the carbonyl of F1 is not involved in a hydrogen bond, L2 is displaced, and the ɑ1 helix begins with G4 [65]. The only crystal structure containing the HIV-1 IN_NTD_ (PDB: 1K6Y) [66] consists of a two-domain truncated form (NTD–CCD) also purified using an N-terminal affinity tag and subsequent thrombin cleavage, leaving 3 residues (G-S-H-) preceding F1 [43, 66]. In this case as well, the ɑ1 helix is shortened, suggesting that the extra N-terminal residues might be disrupting native folding of the ɑ1 helix.

Four NTDs in two structurally distinct positions exist in the HIV-1 core intasome complex cryo-EM structures determined with Sso7d-IN [23, 24]. One NTD, positioned close to the viral DNA and the CCD responsible for catalysis, forms NTD–NTD interactions in the dodecameric HIV-1 intasome and the hexadecameric MVV intasome [25]. The ɑ1 helix of this NTD is shortened in the first HIV-1 tetrameric intasome structure where it begins with Asp 3 [24]. The ɑ1 helix is extended in four of five recent intasome structures, with only one structure showing partial disruption [23]. The second NTD does not interact with the viral DNA and is distant from the active site. This NTD does not form NTD–NTD interactions in dodecameric or hexadecameric intasomes and shows a range of ɑ1 helical structures: disordered, partially unstructured, and extended [23]. Intasomes of a closely-related simian immunodeficiency virus were prepared with IN purified with an N-terminal affinity tag and subsequent human rhinovirus 3C protease cleavage, leaving 3 residues (G-P-G-) preceding F1 [22]. The NTDs in these structures show extended ɑ1 helices.

In this paper, we report a purification scheme of wild type IN with phenylalanine as the N-terminal residue (IN 1F), and associated alterations in the N-terminal structure and IN function. IN 1F was purified with an N-terminal affinity tag, which, when removed, leaves phenylalanine at position 1. We report a two-domain NTD–CCD crystal structure of IN 1F that shows a continuous helical fold beginning with the backbone carbonyl of F1, in contrast to the existing IN GSH_NTD–CCD_ structure [66]. IN 1F also shows greater concerted integration activity in vitro compared to IN GSH and IN MF. IN 1F is altered in its sensitivity to inhibitors, showing decreased sensitivity to the strand-transfer inhibitor raltegravir and increased sensitivity to allosteric integrase inhibitors (ALLINIs). Biophysical characterization reveals that IN 1F has oligomeric properties distinct from previously studied recombinant IN constructs. We propose that HIV-1 IN 1F more closely recapitulates the structure and functions of IN found in authentic HIV infection.

## Methods

### Construction of IN expression vectors

The NL4-3 HIV-1 IN coding sequence was amplified by PCR, fused to an N-His7-Flag-Sumo tag using 4-primer pcr, and cloned into a pCDFDuet expression vector. The fusion junction contains the sequence “G–G–F”, where cleavage by the SUMO protease Ulp1 occurs after the second glycine, liberating IN with a phenylalanine at position 1. IN GSH and IN MF were created by insertion of additional codons preceding the native phenylalanine by inverse PCR (IN GSH) or site-directed mutagenesis (IN MF). IN 1F_NTD–CCD_^F185K, W131D, F139D^ was constructed by truncation of the full-length construct and insertion of a synthetic cassette containing the amino acid substitutions. The lens epithelium derived growth factor (LEDGF) integrase binding domain (IBD) (residues 347–471) was cloned into a pETDuet expression vector with the Mxe intein, a chitin binding domain, and a His6 tag as previously described [67].

### Protein expression and purification

IN constructs were expressed as previously described with some modification [61, 64, 67–69]. Expression plasmids were transformed into *E. coli* BL21(DE3) and grown in 800 mL of 2×YT at 37 °C to an optical density of 1.8–2.2. Expression was induced by addition of isopropyl-β-d-1-thiogalactopyranoside (IPTG) and allowed to continue for 5 h at 20 °C. Bacteria were then pelleted and frozen at − 80 °C.

Full-length IN constructs were purified as described previously [64, 67, 69]. Briefly, lysates were loaded onto nickel-nitrilotriacetic acid resin (Qiagen). Fusion proteins were eluted with 20 mM HEPES–NaOH pH 7.5, 1 M NaCl, 7 mM 3-[(3-cholamidopropyl)dimethylammonio]-1-propanesulfonate (CHAPS), 10 µM ZnOAc_2_, 5 mM β-mercaptoethanol, and 250 mM imidazole. Fusion proteins were liberated from IN by overnight cleavage with the SUMO protease Ulp1 (Life Sensors) at 4 °C, with simultaneous dialysis against 20 mM HEPES–NaOH pH 7.5, 1 M NaCl, 7 mM CHAPS, 10 µM ZnOAc_2_, and 5 mM β-mercaptoethanol. The affinity tag was separated from IN by a second nickel-nitrilotriacetic acid purification step and further purified using a Superdex 75 HiLoad 16/60 column (GE Healthcare) at room temperature, eluted isocratically in 20 mM HEPES–NaOH pH 7.5, 1 M NaCl, 7 mM CHAPS, 10 µM ZnOAc_2_, and 2 mM dithiothreitol (DTT). IN 1F_NTD–CCD_^F185K, W131D, F139D^ was lysed in 50 mM sodium/potassium phosphate pH 7.0, 500 mM NaCl, 2 mM β-mercaptoethanol, and 10 mM imidazole, loaded onto nickel-nitrilotriacetic acid resin, and eluted with 50 mM sodium/potassium phosphate pH 7.0, 500 mM NaCl, 2 mM β-mercaptoethanol, and 250 mM imidazole. The affinity tag was liberated from IN by overnight cleavage with the SUMO protease Ulp1, with simultaneous dialysis against 20 mM sodium/potassium phosphate pH 7.0, 500 mM NaCl, and β-mercaptoethanol. The affinity tag was separated from IN by a second nickel-nitrilotriacetic acid purification step and further purified using a Superdex 75 HiLoad 16/60 column (GE Healthcare) at room temperature, eluted isocratically in 20 mM HEPES–NaOH pH 7.5, 500 mM NaCl, and 2 mM β-mercaptoethanol. IN was concentrated at 4 °C in an Amicon Ultra-15 (Millipore), glycerol was added to a final concentration of 10% (w/v), and aliquots were flash-frozen in liquid nitrogen for storage at − 80 °C.

LEDGF IBD was purified using nickel-nitrilotriacetic acid (Qiagen) and chitin (New England Biolabs) resins. After fusion proteins were liberated by intein cleavage in 50 mM DTT overnight at 4 °C, LEDGF IBD preparations were further purified using a Superdex 75 HiLoad 16/60 column (GE Healthcare) at room temperature, eluted isocratically in 20 mM HEPES–NaOH pH 7.0, 1 M NaCl, 7 mM CHAPS, 10 µM ZnOAc_2_, and 10 mM β-mercaptoethanol. The LEDGF IBD was concentrated at 4 °C in an Amicon Ultra-15 (Millipore), glycerol was added to a final concentration of 10% (w/v), and aliquots were flash-frozen in liquid nitrogen for storage at − 80 °C.

### Crystallization and structure determination

Crystals were grown by vapor diffusion as previously described [66]. Briefly, 4 μL of protein at 5–10 mg/mL in 0.5 M NaCl, 20 mM HEPES pH 7.5, 100 µM ZnCl_2_, 5% (w/v) glycerol, and 5 mM DTT was mixed with 4 μL of reservoir solution containing 0.7 M NaH_2_PO_4_, 1.0 M K_2_HPO_4_ and 0.1 M acetate pH 4.6. Two crystal forms were observed, flat hexagons and long tetragonal crystals, with only the latter exhibiting high resolution diffraction. Crystals were cryo-protected in 0.8 M NaH_2_PO_4_, 1.2 M K_2_HPO_4_, 0.2 M NaCl, and 20% glycerol and flash-frozen in liquid nitrogen. Diffraction data was collected at 100 K using an Eiger 9M pixel-array detector on beamline 17-ID-1 (AMX) at Brookhaven National Laboratory [70, 71].

Diffraction data were reduced with *DIALS* [72]. Molecular replacement, refinement, and the generation of simulated annealing omit maps were carried out in *Phenix* [73]. The structure was solved by molecular replacement using 1K6Y as a search model. The asymmetric unit contained four monomers (each containing a Zn^2+^, K^+^, and phosphate ion) and 226 waters. The structure was refined to a R and R_free_ of 22.5% and 25.3%, respectively. Molecular models were visualised with *Pymol* [74] and secondary structure was analyzed with *Define Secondary Structure of Proteins (DSSP)* [75, 76].

### Integrase 3′-processing assay

The 3′-processing assay was adapted from those described previously [77, 78]. HIV integrase at 60 μM in 20 mM HEPES–NaOH pH 7.5, 1 M NaCl, 7 mM CHAPS, 10 mM DTT, and 10 μM Zn(OAc)_2_ was diluted to a final assay concentration of 400 nM with 20 mM HEPES–NaOH pH 7.5, 100 nM Alexafluor 488-labeled LTR substrate, 50 mM NaCl, 10 mM MgCl_2_ or MnCl_2_, 10 μM Zn(OAc)_2_, and 10 mM DTT. Final assay conditions were identical for IN 1F, IN GSH, and IN MF. Unprocessed U5 LTR substrates with a 3′ Alexafluor 488 *N*-hydroxysuccinimide (NHS) ester label were prepared by annealing the following oligonucleotides (Integrated DNA Technologies):

- 5′-ACCCTTTTAGTCAGTGTGGAAAATCTCTAGCAGT-Alexa488-3′
- 5′-ACTGCTAGAGATTTTCCACACTGACTAAAAGGGT-3′.

Reactions were incubated at 37 °C. SDS was added to a final concentration of 0.25% to stop the reaction and liberate cleaved dinucleotide. After 15 min, fluorescence polarization was analyzed with a plate reader (Victor 3V, Perkin Elmer). Significance was evaluated by two-way ANOVA with P values reported from Tukey’s multiple comparisons test. Data analysis was carried out in *Prism* (GraphPad).

### Integrase strand-transfer assay

The strand-transfer assay was adapted from those described previously [48, 61, 79, 80].

HIV integrase at 60 μM in 20 mM HEPES–NaOH pH 7.5, 1 M NaCl, 7 mM CHAPS, 10 mM DTT, and 10 μM Zn(OAc)_2_ was diluted to a final assay concentration of 3 μM with 20 mM HEPES–NaOH pH 7.5, 0.5 μM Alexafluor 488-labeled LTR substrate, 0.5 μM LEDGF IBD, 50–250 mM NaCl, 10 mM MgCl_2_ or MnCl_2_, and 10 μM Zn(OAc)_2_. Final assay conditions were identical for IN 1F, IN GSH, and IN MF. Processed U5 LTR substrates with a 5′ Alexafluor 488 *N*-hydroxysuccinimide (NHS) ester label were prepared by annealing the following oligonucleotides (Integrated DNA Technologies):

- 5′-Alexa488-ACCCTTTTAGTCAGTGTGGAAAATCTCTAGCA-3′
- 5′-ACTGCTAGAGATTTTCCACACTGACTAAAAGGGT-3′.

After 30 min at 37 °C, 15 nM pUC19 plasmid was added. Reactions were carried out for 1–4 h at 37 °C, then quenched using 0.5% SDS, 15 mM EDTA, and 1 mg/mL proteinase K for 30 min at 37 °C. Reaction products were separated on 1.5% agarose gels in Tris-acetate buffer and imaged using a Typhoon (Amersham) imager. Gels were then stained with ethidium bromide and imaged using a Gel Doc (Bio-Rad) imager. Reaction products were quantified by *ImageJ* and data analysis was carried out in *Prism* (GraphPad). Significance was evaluated by two-way ANOVA with P values reported from Tukey’s multiple comparisons test. Dose–response curve fits were performed in *Prism* (GraphPad) using a three-parameter logistic regression with the Hill slope fixed at − 1. The integrase inhibitor raltegravir was a gift from Merck.

### Aggregation assay for ALLINIs

Assays were performed as previously described [61, 63] with some modification. Final reaction conditions were 20 mM HEPES–NaOH pH 7.5, 15 μM IN, 250–1000 mM NaCl, 7 mM CHAPS, and 30 μM ALLINI. The ALLINIs BI-224436, BI-D, and CX04328 (HIV-1 integrase inhibitor 2) were purchased from MedChemExpress and resuspended in DMSO. Turbidity was measured after 20 min as the absorbance of the reaction solution at 405 nm in a plate reader (Victor 3V, Perkin Elmer). Significance was evaluated by two-way ANOVA with P values reported from Tukey’s multiple comparisons test.

### Size-exclusion chromatography in-line with multi-angle light scattering (SEC-MALS)

Absolute molecular weights were determined by multi-angle light scattering coupled with refractive interferometric detection (Wyatt Technology Corporation) and a Superdex 200 10/300 column (GE Healthcare) at 25 °C equilibrated in 20 mM HEPES–NaOH pH 7.5, 500 mM–1 M NaCl, 7 mM CHAPS, 10 μM ZnOAc_2_, and 10 μM β-mercaptoethanol, as previously described [64].

### Sedimentation velocity analytical ultracentrifugation (SV-AUC)

SV-AUC experiments were performed at 25 °C with an XL-A analytical ultracentrifuge (Beckman-Coulter) and a TiAn60 rotor with two-channel charcoal-filled epon centerpieces and quartz windows. Experiments were performed in 20 mM HEPES–NaOH pH 7.5, 1 M NaCl, 7 mM CHAPS, 10 μM ZnOAc_2_, and 10 μM β-mercaptoethanol. Complete sedimentation velocity profiles were collected every 30 s for 200 boundaries at 40,000 rpm. Data were fit using the *c(s)* distribution model of the Lamm equation as implemented in the program *SEDFIT* [81]. After optimizing meniscus position and fitting limits, the sedimentation coefficients and best-fit frictional ratio (*f/f*_*0*_) were determined by iterative least squares analysis. Sedimentation coefficients were corrected to *s*_*20,w*_ based on the calculated solvent density (ρ) and viscosity (η) derived from chemical composition by the program *SEDNTERP* [82].

### Sedimentation equilibrium analytical ultracentrifugation (SE-AUC)

SE-AUC experiments were performed with an XL-A analytical ultracentrifuge (Beckman-Coulter) and a TiAn60 rotor with two-channel charcoal-filled epon centerpieces and quartz windows. Data were collected at 4 °C with detection at 280 nm at multiple concentrations in 20 mM HEPES–NaOH pH 7.5, 1 M NaCl, 7 mM CHAPS, 10 μM ZnOAc_2_, and 10 μM β-mercaptoethanol. Analyses were carried out using global fits to data acquired at multiple speeds for each concentration with strict mass conservation using the program *SEDPHAT* [83]. Error estimates for equilibrium constants were determined from a 1000-iteration Monte Carlo simulation. The partial specific volume ($\overline{v}$), solvent density (ρ), and viscosity (η) were derived from chemical composition by *SEDNTERP* [82]. SE-AUC data are summarized in Table 2.

## Results

### Cloning and purification of HIV-1 integrase with a native N-terminus

To determine the biochemical and structural properties of HIV-1 IN with a phenylalanine at the N-terminus, we cloned NL4-3 IN into an expression vector containing an N-terminal His7-FLAG-SUMO tag immediately preceding F1. The SUMO protease Ulp1 cleaves at a G–G–/-X motif (with the cleavage site indicated by/, with X being any residue except proline) [84]. This allows for purification of wild-type IN with a native N-terminus (“IN 1F”) by Ulp1 cleavage at the sequence G–G–/-F (Additional file 1: Figure S1). To compare to IN with a non-native N-terminus, we inserted additional N-terminal residues preceding F1. IN GSH contains the three residues (G-S-H) that remain after thrombin cleavage, as used to determine the structure of IN GSH_NTD–CCD_ (PDB: 1K6Y) [66], and IN MF contains an N-terminal methionine found in constructs commonly used for bacterial overexpression [61, 63, 64]. A nickel-affinity step captures Ulp1 and the cleaved affinity tag and subsequent size-exclusion chromatography yields a highly pure final product (Additional file 1: Figure S1).

### Crystallization of an IN 1F NTD–CCD derivative

**Fig. 1:**
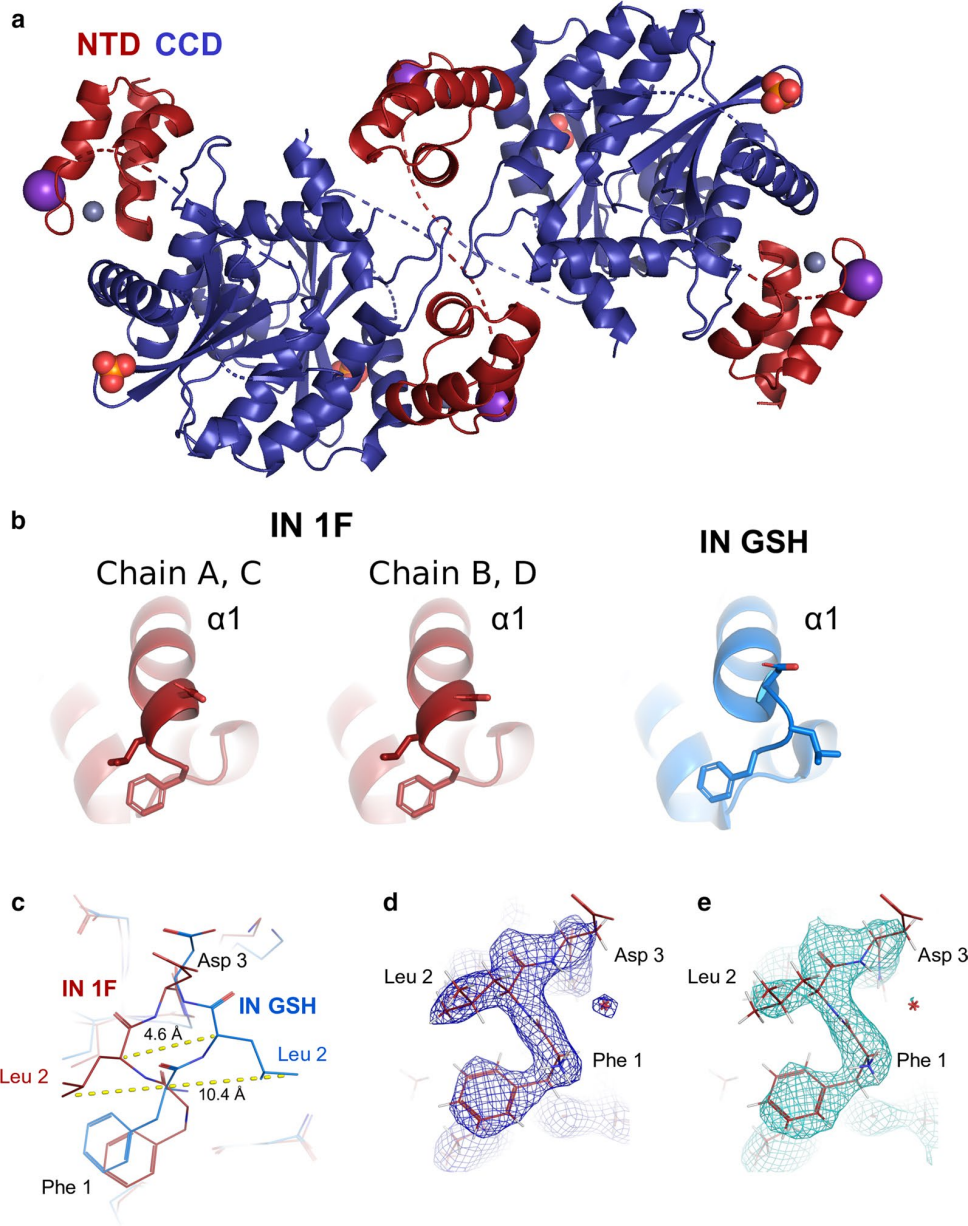
Structure of HIV-1 integrase with a native N-terminus (PDB 6VRG). **a** Structure of IN 1F_NTD–CCD_. NTDs are colored in red and CCDs are colored in blue. Zn^2+^ (grey), K^+^ (purple), and phosphate (orange and red) atoms are shown as spheres. **b** Comparison of the ɑ1 helix between IN 1F and IN GSH (PDB 1K6Y). The IN 1F NTD adopts a helical structure starting from the carbonyl of F1. The IN GSH NTD shows a disruption of the ɑ1 helix. **c** View highlighting differences between IN 1F and IN GSH at the N-terminus, with a deviation of 4.6 Å in the peptide backbone at L2 and a 10.4 Å deviation in side chain position. This change is accompanied by a flip of ~ 180° in the orientation of the N-terminus. 2Fo-Fc electron density (**d**) and simulated annealing omit (**e**) maps contoured at 1.5 σ unambiguously demonstrate the N-terminal structure of IN 1F

To investigate structural differences between IN 1F and IN GSH, we created an IN 1F_NTD–CCD_ construct containing the same solubility-enhancing substitutions (W131D, F139D, and F185K) used to determine the structure of IN GSH_NTD–CCD_ [66]. Affinity purification, Ulp1 cleavage, and size-exclusion chromatography yielded a highly pure final product (Additional file 1: Figure S1) that readily crystallized as described previously [66]. The structure was solved by molecular replacement, using the existing NTD–CCD structure (PDB: 1K6Y) as a search model. Four copies of both the NTD and the CCD were present in the asymmetric unit (Fig. 1a), with the inter-domain linker (residues 47–55) unresolved in the electron density. In the structure of IN GSH_NTD–CCD_, each NTD is assigned to a “distal” position relative to the CCD (Additional file 2: Figure S2). However, in the crystal structure of the HIV-2 IN_NTD–CCD_ complexed with the lens epithelium derived growth factor (LEDGF) integrase binding domain (IBD) (PDB: 3F9K) [85], the interdomain linker is well-defined in the electron density, placing the NTDs in a “proximal” position relative to the CCD (Additional file 2: Figure S2). This is also the favored position for the NTDs in small angle X-ray scattering (SAXS) analysis of IN NTD–CCD coexpressed with the LEDGF IBD [64]. In the IN 1F_NTD–CCD_ structure, the unresolved 10-residue linker would be long enough to span the unobstructed distance of 28.7–31.8 Å to position the NTDs in a “proximal” position. We have therefore defined the NTDs in the “proximal” orientation relative to the CCDs, as observed in the HIV-2 IN_NTD–CCD_ structure (Fig. 1a). Crystallographic statistics are summarized in Table 1.

**Table 1:** Crystallographic statistics

Data collection	Overall (highest shell)
Space group	P4_3_2_1_1
Unit cell dimensions	102.919 Å × 102.919 Å × 279.203 Å; 90°, 90°, 90°
Resolution range (Å)	96.567–2.40
R_merge_	0.558 (1.619)
R_p.i.m._	0.110 (0.318)
CC_1/2_	0.990 (0.289)
Multiplicity	26.6 (26.6)
I/σ	8.1 (1.6)
Completeness (%)	100 (100)
Refinement	
Reflections (work)	58,492
Reflections (free)	1169
R_work_	22.5%
R_free_	25.3%
Protein atoms	12,003
Ligand/ion atoms	28
Water molecules	226
R.m.s. bonds (Å)	0.004
R.m.s. angles (°)	0.589
Ramachandran plot (%)	
Favored	98.49
Allowed	1.51
Outliers	0.00

### Structure of the IN 1F NTD–CCD construct

The overall structure of IN 1F_NTD–CCD_ is highly similar to IN GSH_NTD–CCD_ (global RMSD: 0.90 Å). A phosphate ion is found near the active site of each CCD. Each copy of the NTD folds into a 3-helix motif coordinating a Zn^2+^ ion with residues H12, H16, C40, and C43. A potassium ion is coordinated by the carbonyl oxygens of V37, A38, C40, C43. Close inspection of the N-terminus reveals differences between IN 1F and IN GSH (Fig. 1b). In the asymmetric unit of IN 1F, two secondary structures are observed at the N-terminus. The ɑ1 helix in chains A and C begins as a hydrogen-bonded turn at the backbone carbonyl of F1, while in chains B and D, a canonical alpha helix begins at the backbone carbonyl of F1 (Fig. 1b, Additional file 3: Figure S3). In IN GSH, the ɑ1 helix does not begin until D3 due to a shift in the L2 side chain by ~ 10 Å, accompanied by a ~ 4.6 Å displacement of the peptide backbone at L2 (Fig. 1b, c, Additional file 3: Figure S3). F1 is in a similar position in IN 1F and IN GSH, where it caps a hydrophobic core in the NTD made up of I5, L28, P29, and V32. The N-terminal amino group also differs between these two structures due to the peptide backbone displacement at L2. In IN GSH, the N-terminal amino group is oriented toward the C-terminal end of the ɑ2 helix, whereas in IN 1F, it is flipped ~ 180° and oriented toward the ɑ3 helix of a neighboring NTD. The same NTD–NTD interface is observed in dodecameric HIV-1, hexadecameric MVV, and SIV intasome structures [22–25], and the NTDs modeled at this position adopt extended ɑ1 helical structures in four of six structures (Additional file 3: Figure S3). The NTDs that do not form an NTD–NTD interface show a variety of structures: disordered, partially unstructured, and extended (Additional file 3: Figure S3). Difference maps and simulated annealing omit maps calculated around the N-terminus of each protomer of the IN 1F_NTD–CCD_ structure confirmed the observed differences between the N-termini of IN 1F and IN GSH (Fig. 1d, e).

### Activity of IN 1F in vitro

**Fig. 2:**
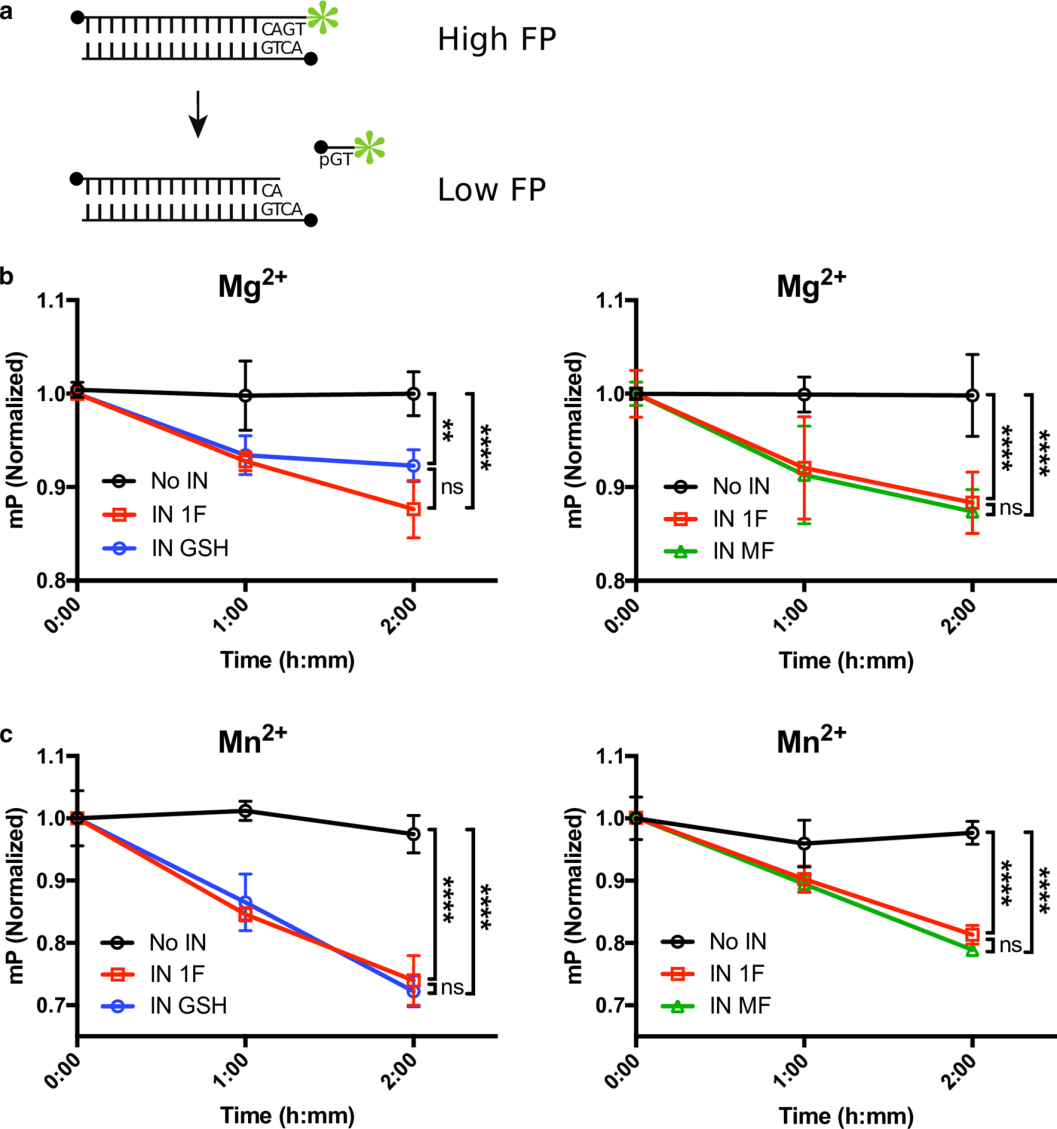
3′-processing activity in vitro. **a** Diagram of 3′-processing fluorescence polarization assay. The double stranded oligo containing a 3′ fluorescent label exhibits high fluorescence polarization. Cleavage and release of the terminal dinucleotide causes a decrease in fluorescence polarization. 5′-ends are designated by filled circles and the fluorophore is designated by the green star. **b** 3′-processing activity of IN 1F compared to IN GSH (left) and IN 1F compared to IN MF (right) in the presence of Mg^2+^. **c** 3′ processing activity of IN 1F compared to IN GSH (left) and IN 1F compared to IN MF (right) in the presence of Mn^2+^. Data are plotted as mean ± SD. ** Denotes P < 0.01 and **** denotes P < 0.0001

IN carries out two catalytic functions, 3′-processing and strand transfer, which can be replicated in vitro using fluorescently-labeled oligonucleotides that mimic the viral long terminal repeat (LTR). To assay 3′-processing, we used a 3′-fluorescently-labeled double-stranded oligonucleotide mimicking the viral LTR to monitor release of the terminal dinucleotide (5′-GT-3′) using fluorescence polarization [77]. The unprocessed oligonucleotide emits highly polarized fluorescence. Upon cleavage by IN, the released dinucleotide emits fluorescence with low polarization. In the presence of Mg^2+^ and Mn^2+^, IN 1F, IN GSH, and IN MF showed similar 3′-processing activities (Fig. 2).

**Fig. 3:**
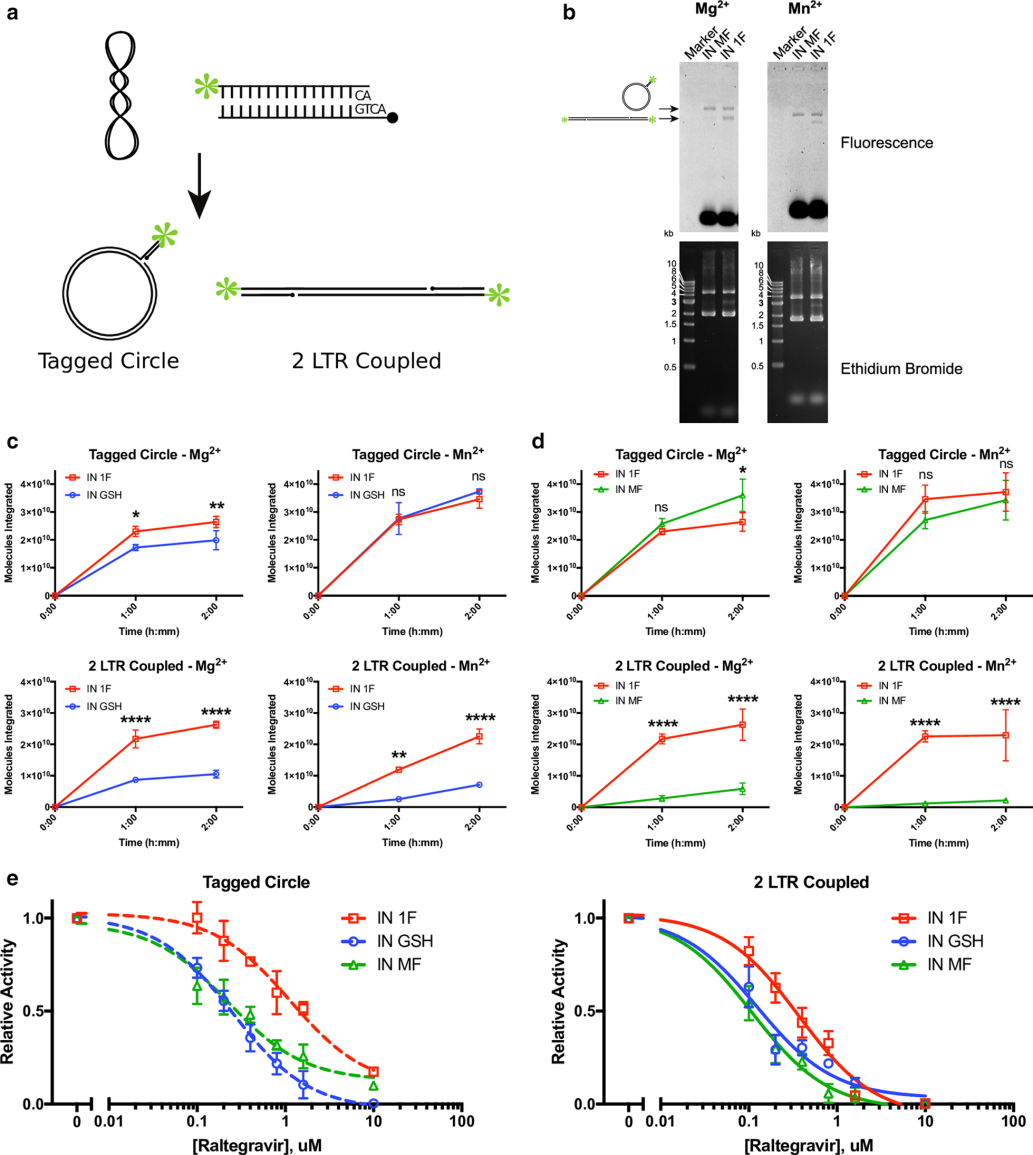
Strand transfer activity in vitro. **a** Diagram of gel-based strand transfer assay. A pre-processed double-stranded oligo containing a 5′ fluorescent label is integrated into a supercoiled target plasmid (pUC19) in the presence of integrase and cofactor (Mg^2+^ or Mn^2+^). Single strand integration results in the formation of a tagged circle product accompanied by the relaxation of supercoiling. Concerted integration results in the formation of a 2 LTR coupled product, accompanied by the linearization of the plasmid. Reaction products are separated by agarose gel electrophoresis. **b** Example gels of IN 1F and IN MF strand transfer activity. The slowest-migrating band represents single strand integration events (tagged circle) with the linearized concerted integration product (2 LTR coupled) migrating further. The supercoiled target plasmid is visible in the ethidium bromide stain as the furthest-migrating band. Unintegrated fluorescent oligo is observed at the bottom of the gel. **c** Quantification of IN 1F and IN GSH strand transfer activity. Single strand integration events are shown in the top panels and concerted integration events are shown in the bottom panels. IN 1F shows greater concerted integration activity than IN GSH in the presence of either Mg^2+^ or Mn^2+^. **d** Quantification of IN 1F and IN MF strand transfer activity. Single strand integration events are shown in the top panels and concerted integration events are shown in the bottom panels. IN 1F shows greater concerted integration activity than IN MF in the presence of either Mg^2+^ or Mn^2+^. **e** Inhibition of strand transfer activity by raltegravir. Raltegravir more potently inhibits both the single strand (left) and concerted integration (right) activity of IN GSH and IN MF as compared to IN 1F. Data before normalization are plotted in Additional file 5: Figure S5. Data are plotted as mean ± SD of 3 replicates. * Denotes P < 0.05, ** denotes P < 0.01, and **** denotes P < 0.0001

To assay strand transfer activity, we used 5′-fluorescently-labeled oligonucleotides mimicking the viral LTR and a supercoiled plasmid mimicking nucleosomal DNA (Fig. 3a). Concerted integration of two viral LTRs by IN results in linearization of the supercoiled plasmid and incorporation of the fluorescent label. Strand-transfer activity in the presence of Mg^2+^ and Mn^2+^ was influenced by NaCl concentration, with the highest level of concerted integration occurring at 150 mM NaCl in the presence of Mg^2+^ and 200–250 mM NaCl in the presence of Mn^2+^ (Additional file 4: Figure S4). In identical assay conditions, IN 1F showed superior concerted integration activity, resulting in the formation of 2 LTR coupled products, as compared to IN GSH and IN MF at all time points measured (Fig. 3b, c). This difference was observed in the presence of either Mg^2+^ or Mn^2+^.

A partial reaction, the integration of a single LTR oligo, results in relaxation of the supercoiled plasmid and incorporation of the fluorescent label. Quantification of the fluorescently tagged, relaxed-circular plasmid indicates single-ended integration activity. Single-end activity, resulting in the formation of tagged circle products, was not improved by IN 1F as compared to IN GSH or IN MF (Fig. 3c).

Treatment with the strand transfer inhibitor raltegravir more potently inhibited both the single-strand and concerted integration activity of IN GSH and IN MF as compared to IN 1F (Fig. 3d, Additional file 5: Figure S5). The IC_50_ for inhibiting concerted integration was 125 nM (95% CI 83–186 nM), 109 nM (95% CI 83–142 nM), and 370 nM (95% CI 270–508 nM) for IN GSH, IN MF, IN 1F, respectively. The IC_50_ for inhibiting single-strand integration was 253 nM (95% CI 202–318 nM), 223 nM (95% CI 141–356 nM), and 1.13 µM (95% CI 0.73–1.8 µM) for IN GSH, IN MF, and IN 1F, respectively.

### Response of IN 1F and IN MF to ALLINIs

**Fig. 4:**
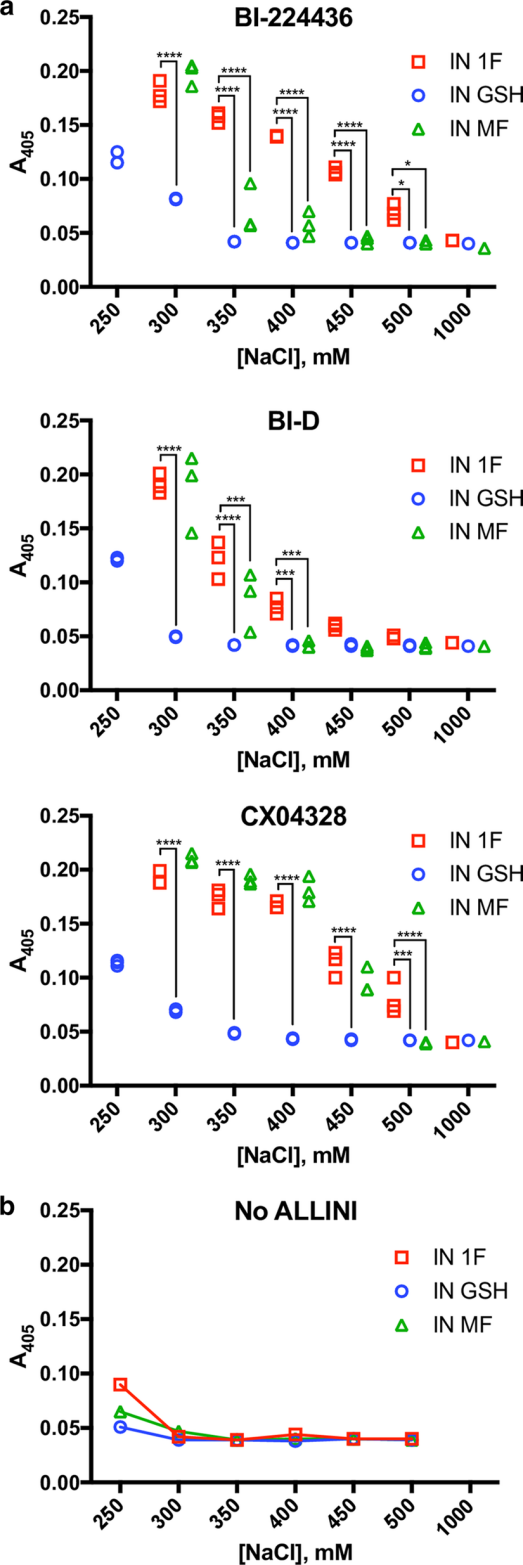
ALLINI-induced aggregation of IN 1F, IN GSH, and IN MF. **a** Aggregation of IN at 15 µM was induced by incubation with 30 µM of the ALLINIs BI-224436, BI-D, or CX04328 at a range of NaCl concentrations for 20 min. Aggregation was measured by light scattering at 405 nm. No aggregation was observed for any ALLINI at 1000 mM NaCl. At intermediate NaCl concentrations, ALLINIs induce aggregation of IN 1F more potently than IN GSH or IN MF. **b** Aggregation in the absence of ALLINIs. No aggregation was observed at NaCl concentrations of 300 mM or greater. Below 300 mM, IN 1F, and, to a lesser extent, IN MF and IN GSH spontaneously aggregate. * Denotes P < 0.05, ** denotes P < 0.01, *** denotes P < 0.001, **** denotes P <* 0.0001*

The allosteric inhibitors of integrase (ALLINIs) [86–88] are a class of small molecule inhibitors that block the interaction of IN with LEDGF and cause aberrant aggregation of IN [61, 63, 89]. ALLINIs aggregate recombinant IN in vitro, causing turbidity that can be measured by light scattering [61, 63, 64]. Using this approach, we measured the sensitivity of IN 1F, IN GSH, and IN MF to ALLINIs (Fig. 4). ALLINI-induced aggregation is NaCl-dependent, so we tested aggregation at NaCl concentrations from 250 mM to 1 M. At 1 M NaCl, no aggregation was observed by the ALLINIs BI-224436 [87], BI-D [90], or CX04328 (Compound 6 from Christ et al. [86]). At NaCl concentrations where ALLINI-induced aggregation was observed, ALLINIs induced equal or greater aggregation of IN 1F as compared to IN GSH or IN MF. BI-224436, BI-D, and CX04328 aggregated IN 1F more than IN GSH at 300–500 mM NaCl, 300–400 mM NaCl, and 300–500 mM NaCl, respectively. Significant ALLINI-induced aggregation of IN GSH was only observed at 250 mM NaCl, where IN 1F was observed to aggregate in the absence of ALLINI. BI-224436, BI-D, and CX04328 aggregated IN 1F more than IN MF from 350 to 500 mM NaCl, 350–400 mM, and 500 mM NaCl, respectively. At lower NaCl concentrations, ALLINIs induced aggregation of IN 1F and IN MF to an equal extent.

### Solution properties of IN with a native N-terminus

**Fig. 5:**
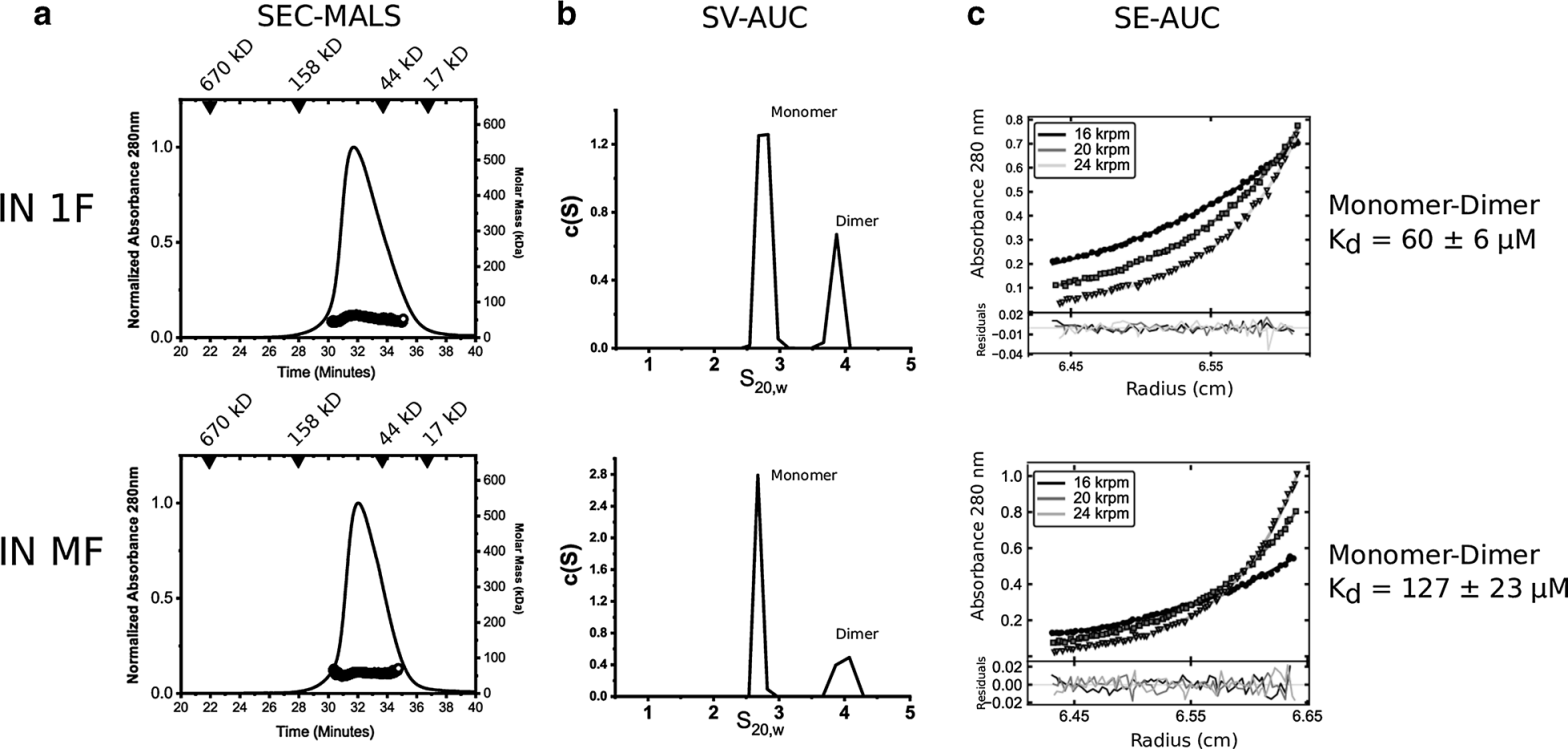
Biophysical analysis of IN 1F and IN MF. **a** SEC-MALS analysis of IN 1F and IN MF. Both the M_w_ (weight-average molecular mass) from multiangle light scattering and retention times are consistent with mixtures of monomers and dimers for both IN 1F and IN MF (expected MW of monomer: 32 kDa). **b** Sedimentation velocity analysis of IN 1F and IN MF shows distinct populations of monomer and dimer in solution. c(S) distributions derived from the fitting of the Lamm equation are shown. **c** Sedimentation equilibrium analysis of IN 1F and IN MF indicates the presence of monomers and dimers in solution at 4 °C. Globally fit radial distributions for 8.9 μM (IN 1F) and 6 μM (IN MF) in a monomer–dimer model are shown. Table 2 provides the association properties derived from this analysis

One possible explanation for the improved activity of IN 1F is that folding associated with the native N-terminal sequence changes the oligomerization state. To investigate this possibility, we analyzed IN 1F and IN MF by size-exclusion chromatography in line with multi-angle light scattering (SEC-MALS) to determine the oligomeric state in solution. Both IN 1F and IN MF showed mass profiles consistent with a monomer–dimer transition (32–64 kD, expected MW of monomer: 32 kD) at eluted concentrations of ~ 8–10 µM, as well as retention times consistent with a mixture of monomers and dimers (Fig. 5a). Sedimentation velocity analytical ultracentrifugation (SV-AUC) experiments performed at similar concentrations and temperatures confirmed the presence of monomers and dimers with the presence of two discrete species at ~ 2.8 S and ~ 4 S, respectively (Fig. 5b). Sedimentation equilibrium analytical ultracentrifugation (SE-AUC) analysis at 4 °C and similar concentrations also confirmed the presence of monomers and dimers, and global fitting of a monomer–dimer equilibrium yielded a K_d_ of 60 ± 6 µM and 127 ± 23 µM for IN 1F and IN MF, respectively (Fig. 5c, Table 2). An attempt to fit a dimer–tetramer equilibrium could only be accomplished with a K_d_ > 1 mM. Therefore, no evidence of tetramers was observed by three biophysical methods, in contrast to prior studies performed under similar conditions with IN expression constructs with an N-terminal methionine [61, 63, 64], N-terminal thrombin [42], or human rhinovirus 3C protease [13, 85] cleavage sequences.

**Table 2:** Properties determined by sedimentation equilibrium analysis

Protein	Concentrations (µM)	Speeds (krpm)	Model fit	Mass^a^ (Da)	K_d_ (µM)	Global reduced χ^2^
IN 1F	8.9, 14.5	16,20,24	M–D	32,330	59.8 ± 6	2.7
IN MF	6.0, 7.9	16,20,24	M–D	32,199	126.7 ± 23	1.3

*M* monomer, *D* dimer

^a^Calculated mass of monomer from sequence

The monomer–dimer behavior of IN 1F in solution also differs from IN with an N-terminal methionine, C-terminal intein cleavage site, and the solubility-enhancing substitution F185H, which exists as a mixture of dimers and tetramers in solution [61, 64] and is replication-competent in virus [91]. Introduction of the F185H substitution into IN 1F (IN 1F^F185H^) resulted in the formation of dimers and a spectrum of higher-order aggregates in solution, as determined by SEC-MALS (Additional file 6: Figure S6). IN 1F^F185H^ retained similar 3′-processing activity as compared to IN 1F, but showed a significant decrease in single strand and concerted strand transfer activity (Additional file 6: Figure S6), indicating the effect of the oligomeric state of IN on strand transfer activity.

## Discussion

In this paper, we report the construction and purification of IN with a native N-terminus (IN 1F). The crystal structure of IN 1F_NTD–CCD_ reveals an extended ɑ1 helix starting with F1, as compared to IN GSH_NTD–CCD_ with a shortened helix. Despite the remainder of the structure showing little to no difference, this change in the N-terminus is sufficient to improve concerted integration activity. In contrast, the 3′-processing and single strand integration activities were not affected. We also observed a change in sensitivity to IN-targeting antiretroviral drugs. IN 1F was less sensitive to the STI raltegravir and more sensitive to ALLINI-induced aggregation. We suggest that IN 1F will be useful in studies of IN function and response to inhibitors in the future.

The zinc finger fold of the NTD is shared with other DNA-binding proteins [92–94], with residues homologous to positions 1–3 in IN located adjacent to the phosphate backbone of DNA. In retroviral intasomes, the NTD binds to the distal viral DNA ends [19, 23–26, 39, 40]. However, unlike other helix-turn-helix binding proteins, the NTD does not insert a helix into the major groove of DNA, and F1 is distant from the phosphate backbone. The effect of the N-terminal disruption in IN GSH and IN MF is unclear, because the change is not expected to disrupt a tetrameric intasome. In the hexadecameric maedi-visna virus intasome, however, two pairs of NTDs are closely oriented head-to-head [25], forming a nearly identical NTD–NTD interface as that observed in the structure of IN 1F and IN GSH_NTD–CCD_ [66]. This hydrophobic dimerization interface would involve significant contributions from F1, in contrast to the dimerization interface of the isolated NTD which mainly involves the ɑ3 helix [3]. Additional N-terminal residues, such as the N-terminal Sso7d-IN fusion, could induce a steric clash [24]. It is possible that such a disruption explains the presence of heterogeneous, poorly resolved higher-order intasomes reported in the cryo-EM studies of HIV-1 Sso7d-IN intasomes [23, 24]. Additionally, disruption of the ɑ1 helix could affect binding to LEDGF, as the NTD cooperates with the CCD in binding LEDGF [13, 85]. Destabilization of the intasome and disruption of the IN-LEDGF interaction are possible explanations for the differences in concerted integration activity and STI sensitivity between IN 1F, IN GSH, and IN MF.

Surprisingly, we found IN 1F to be more potently aggregated by ALLINIs compared to IN GSH and IN MF. ALLINIs cause the formation of open polymers of IN mediated by CCD–CTD interactions [61], and it is not immediately clear how addition of N-terminal residues affects this process. Previously, we have shown that the NTD is dispensable for ALLINI-induced aggregation [63], although others have reported that constructs lacking the NTD are resistant to ALLINI-induced aggregation [95], suggesting that the NTD plays a role in modulating ALLINI-induced aggregation. In multiple structures [13, 66, 85], the NTD interacts with the CCD in a manner expected to clash with the CCD–CTD interactions observed in the ALLINI-induced IN polymer. An effect on competition between the NTD and CTD for CCD binding may explain the difference in ALLINI potency between IN 1F, IN GSH, and IN MF. Recently, IN tetramers have been implicated as the preferred target of ALLINIs [95], but we show that IN 1F, which is a mixture of monomers and dimers in solution, is aggregated by ALLINIs. However, aggregation is NaCl-dependent, and we have not determined the oligomeric state of IN 1F at lower NaCl concentrations. IN GSH remains soluble at NaCl concentrations that lead to aggregation of IN 1F in the absence of ALLINI, demonstrating that additional N-terminal residues can improve solubility. This is consistent with the observation of improved solubility of Sso7d-IN [51] and PFV IN, which harbors an N-terminal extension domain [26, 41]. Additional experiments are needed to determine the details of ALLINI-induced polymer initiation and propagation.

Wild type IN 1F is a mixture of monomers and dimers in solution, which differs from previously reported IN preparations containing substitutions at F185 or additional N-terminal residues which are a mixture of dimers and tetramers [13, 43, 61, 64]. We found that the substitution F185H in the IN 1F background resulted in the formation of higher-order species in solution. NTD–CCD interactions between residues such as E11 and K186 have been shown to be important for tetramerization [13, 95], and we have now shown that modification of the adjacent residue F185 affects oligomerization in the context of a native N-terminus. Notably, the construct used to solve the only HIV IN crystal structure with a naturally-occurring F185, HIV-2 IN_NTD–CCD_ co-expressed with LEDGF IBD, was dimeric in solution [85]. In this structure, the interdomain linker is clearly resolved in the electron density, showing that the NTD contacts the CCD in a “proximal” orientation. This is in contrast to the IN GSH_NTD–CCD_ structure (PDB: 1K6Y) where the interdomain linker is not resolved, short interdomain linkers are assigned, and each NTD is in a “distal” orientation [66]. The interdomain linker is not resolved in our IN 1F_NTD–CCD_ structure, but we favor longer interdomain linkers, positioning each NTD in a “proximal” orientation, as this is the orientation observed in the HIV-2 IN_NTD–CCD_-LEDGF co-crystal structure [85]. Additional work is needed to understand the effect of substitutions at F185 and K186 on NTD–CCD interactions in dimeric forms of IN.

## Conclusions

HIV IN containing a native N-terminus adopts a distinct structural configuration, shows improved activity in vitro, and manifests altered sensitivity to inhibitors. Because it mimics the form of IN produced by proteolytic cleavage in the maturing virion, IN 1F provides an improved reagent for the study of IN activity in vitro and for use in antiviral drug development.

## Supplementary information

**Additional file 1: Figure S1.** a) Purification scheme of IN with a native N-terminus (IN 1F). The poly-histidine (His7) affinity tag allows for capture of fusion proteins on Ni^2+^-NTA resin. Subsequent cleavage by the SUMO protease Ulp1 frees wild type IN with a phenylalanine at position 1. b) Coomassie-stained SDS-PAGE analysis of IN constructs after Ulp1 cleavage and size-exclusion chromatography. Expected protein size is 32 kDa. c) Coomassie-stained SDS-PAGE analysis of IN 1F_NTD-CCD_ with the substitutions F185K, W131D, and F139D that enable crystallization. Expected protein size is 23 kDa.

**Additional file 2: Figure S2.** Models demonstrating two possibilities of NTD domain orientation. IN is depicted as a dimer with both NTDs in either the “distal” or “proximal” orientation. Models are based on PDB structures 1K6Y, 5HOT, and 6VRG.

**Additional file 3: Figure S3.** a) Secondary structure annotation of NTDs (residues 1-50) of IN 1F (PDB: 6VRG), IN GSH (PDB: 1K6Y), and Sso7d-IN (PDB: 5U1C, 6PUT, 6PUW, 6PUY, 6PUZ, and 6V3K) by *DSSP*. H = Alpha Helix, G = 3_10_ Helix, T = Hydrogen bonded turn, and S = Bend. b) Comparison of Sso7d-IN ɑ1 helix structures. Chain A is predicted to form NTD–NTD interactions in dodecameric HIV-1 and hexadecameric MVV intasomes. Chain B is not predicted to form NTD–NTD interactions.

**Additional file 4: Figure S4.** NaCl-dependence of strand transfer activity. a) In the presence of Mg^2+^, IN 1F and IN MF are most active at low NaCl concentrations, with activity disappearing above a NaCl concentration of 200 mM. The highest level of concerted integration activity is observed at 150 mM NaCl. b) In the presence of Mn^2+^, IN 1F and IN MF are most active at NaCl concentrations higher than in the presence of Mg^2+^. The highest level of concerted integration activity is observed at 250 mM NaCl.

**Additional file 5: Figure S5.** Effect of raltegravir on strand transfer activity of IN 1F, IN GSH, and IN 1F. Data are the same as in Fig. 3e but plotted as molecules integrated per minute of single strand (left) and concerted integration (right) without normalization. Data plotted as mean ± SD of 3 replicates.

**Additional file 6: Figure S6.** Biophysical and biochemical characterization of IN 1F^F185H^. a) SEC-MALS analysis of IN 1F^F185H^ shows a mixture of dimers and higher-order aggregates in solution. b) Quantification of fluorescence polarization assay for 3’ processing. IN 1F^F185H^ performs 3’-processing more rapidly than IN 1F, a difference that reaches statistical significance, but has unclear biological relevance. c) Example gel image of results from strand transfer assay in the presence of Mg^2+^. d) Quantification of strand transfer activity. The single strand and concerted strand transfer activity of IN 1F^F185H^ is significantly decreased as compared to wild type IN 1F. Data plotted as mean ± SD of 3 replicates. * denotes P *<* 0.05, *** denotes P *=* 0.0005, **** denotes P *<* *0.0001.*

**Additional file 7: File S7.** Spreadsheet containing the underlying data for Figs. 2, 3, 4, Additional file 5: Figure S5, and Additional file 6: Figure S6.

## Data Availability

All data generated and/or analysed during this study are included in this published article, its supplementary information files, and in the Worldwide Protein Data Bank repository under accession number 6VRG (Additional file 7).

## Abbreviations

ART: Antiretroviral therapy
HIV: Human immunodeficiency virus
IN: Integrase
IN 1F: Integrase with native N-terminal phenylalanine
IN GSH: Integrase with Gly-Ser-His preceding N-terminal phenylalanine
IN MF: Integrase with methionine preceding N-terminal phenylalanine
NTD: N-terminal domain
CCD: Catalytic core domain
CTD: C-terminal domain
PFV: Prototype foamy virus
STI: Strand transfer inhibitor
RSV: Rous sarcoma virus
MMTV: Mouse mammary tumor virus
MVV: Maedi-visna virus
ALLINI: Allosteric integrase inhibitor
LEDGF: Lens epithelium-derived growth factor
IBD: Integrase binding domain
IPTG: Isopropyl-β-d-1-thiogalactopyranoside
CHAPS: 3-[(3-cholamidopropyl)dimethylammonio]-1-propanesulfonate
DTT: Dithiothreitol
AMX: Highly automated macromolecular crystallography
DIALS: Diffraction integration for advanced light sources
DSSP: Define secondary structure of proteins
NHS: *N*-hydroxysuccinimide
SV-AUC: Sedimentation velocity analytical ultracentrifugation
SE-AUC: Sedimentation equilibrium analytical ultracentrifugation
SAXS: Small angle X-ray scattering
LTR: Long terminal repeat
SEC-MALS: Size-exclusion chromatography in line with multi-angle light scattering
