# Supplementary figures and images for: Influence of the amino-terminal sequence on the structure and function of HIV integrase
Source: Retrovirology. 2020 Aug 31;17:28. doi: 10.1186/s12977-020-00537-x (PMC7457537; doi:10.1186/s12977-020-00537-x)

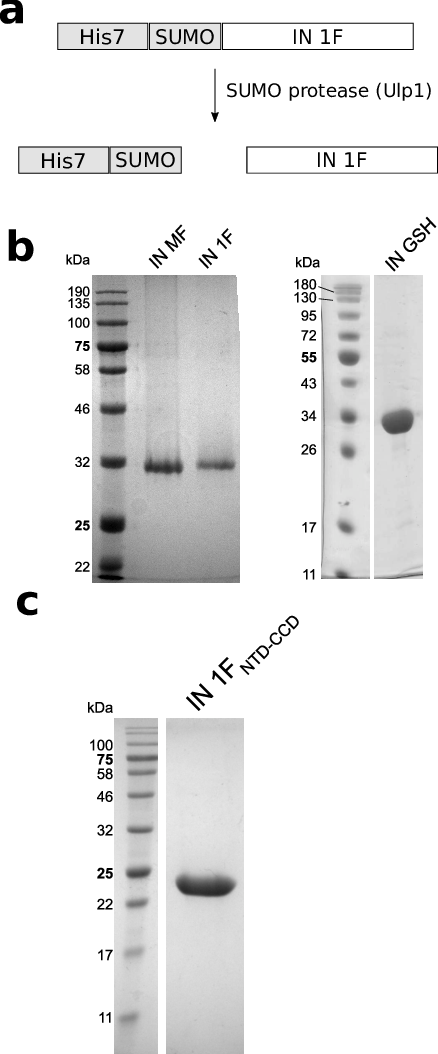

Supplement: Supplementary file 1 — Additional file 1: Figure S1. a) Purification scheme of IN with a native N-terminus (IN 1F). The poly-histidine (His7) affinity tag allows for capture of fusion proteins on Ni2+-NTA resin. Subsequent cleavage by the SUMO protease Ulp1 frees wild type IN with a phenylalanine at position 1. b) Coomassie-stained SDS-PAGE analysis of IN constructs after Ulp1 cleavage and size-exclusion chromatography. Expected protein size is 32 kDa. c) Coomassie-stained SDS-PAGE analysis of IN 1FNTD-CCD with the substitutions F185K, W131D, and F139D that enable crystallization. Expected protein size is 23 kDa. [file 12977_2020_537_MOESM1_ESM.tif]

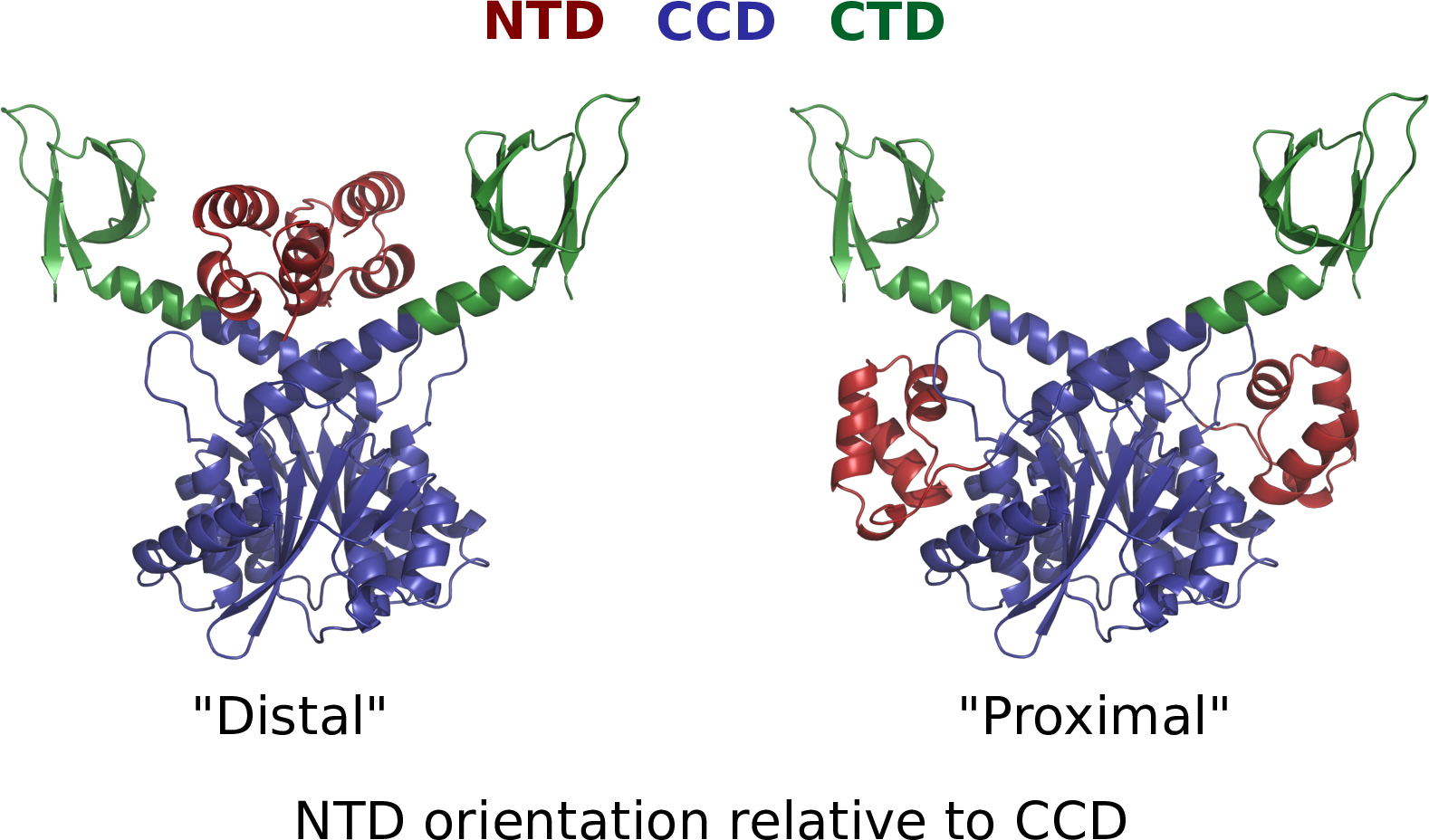

Supplement: Supplementary file 2 — Additional file 2: Figure S2. Models demonstrating two possibilities of NTD domain orientation. IN is depicted as a dimer with both NTDs in either the “distal” or “proximal” orientation. Models are based on PDB structures 1K6Y, 5HOT, and 6VRG. [file 12977_2020_537_MOESM2_ESM.tif]

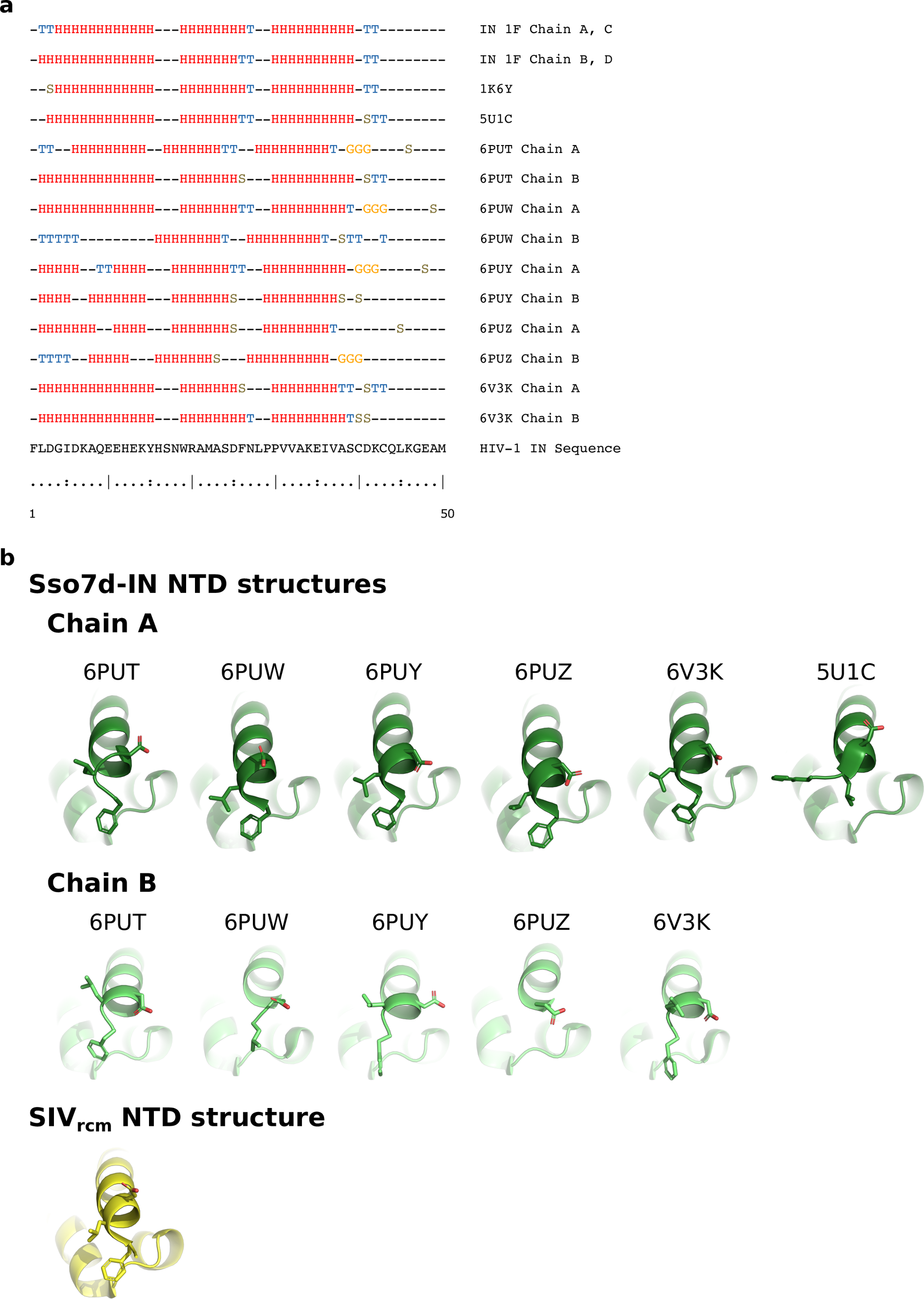

Supplement: Supplementary file 3 — Additional file 3: Figure S3. a) Secondary structure annotation of NTDs (residues 1-50) of IN 1F (PDB: 6VRG), IN GSH (PDB: 1K6Y), and Sso7d-IN (PDB: 5U1C, 6PUT, 6PUW, 6PUY, 6PUZ, and 6V3K) by DSSP. H = Alpha Helix, G = 310 Helix, T = Hydrogen bonded turn, and S = Bend. b) Comparison of Sso7d-IN ɑ1 helix structures. Chain A is predicted to form NTD–NTD interactions in dodecameric HIV-1 and hexadecameric MVV intasomes. Chain B is not predicted to form NTD–NTD interactions. [file 12977_2020_537_MOESM3_ESM.tif]

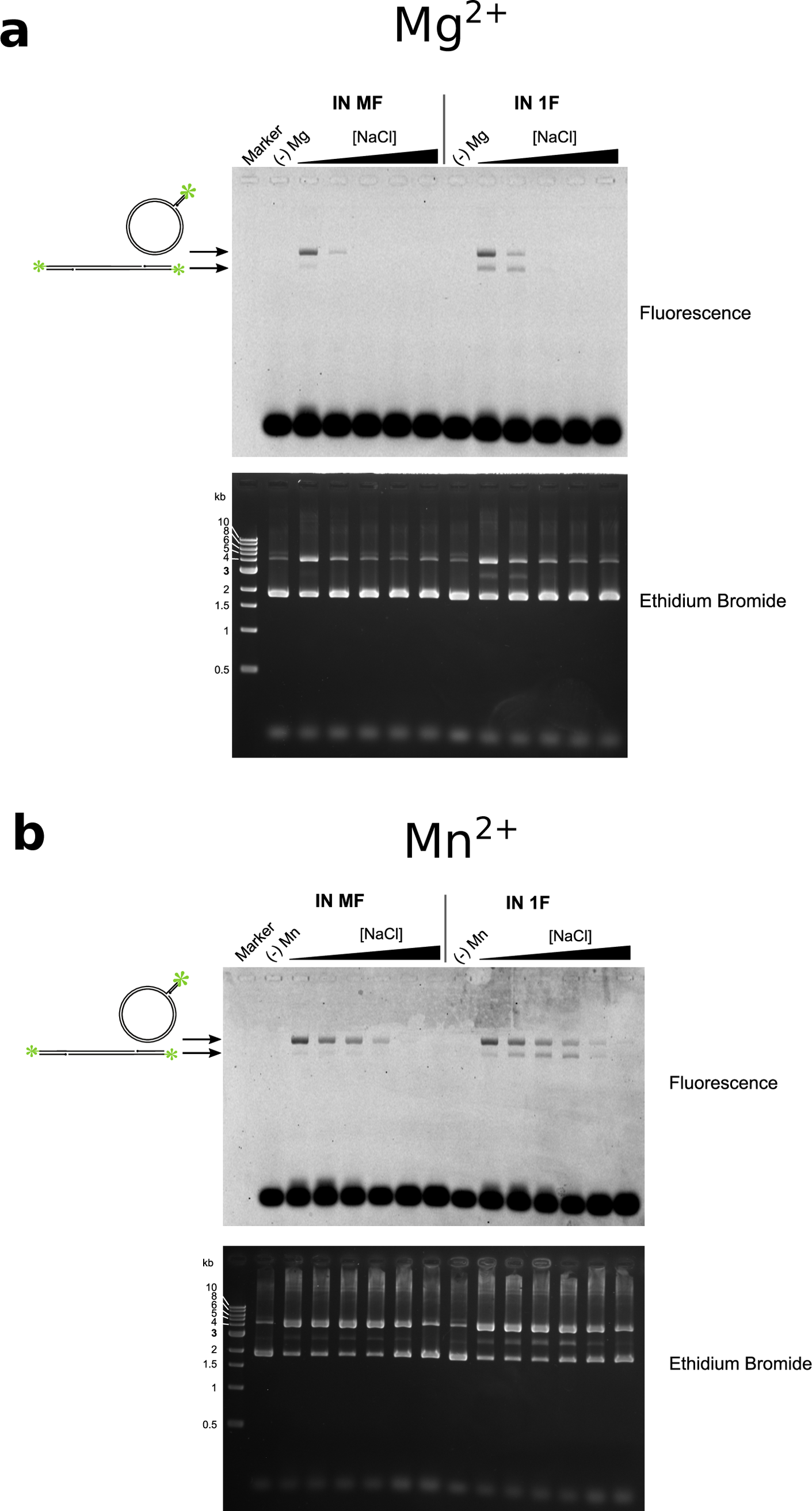

Supplement: Supplementary file 4 — Additional file 4: Figure S4. NaCl-dependence of strand transfer activity. a) In the presence of Mg2+, IN 1F and IN MF are most active at low NaCl concentrations, with activity disappearing above a NaCl concentration of 200 mM. The highest level of concerted integration activity is observed at 150 mM NaCl. b) In the presence of Mn2+, IN 1F and IN MF are most active at NaCl concentrations higher than in the presence of Mg2+. The highest level of concerted integration activity is observed at 250 mM NaCl. [file 12977_2020_537_MOESM4_ESM.tif]

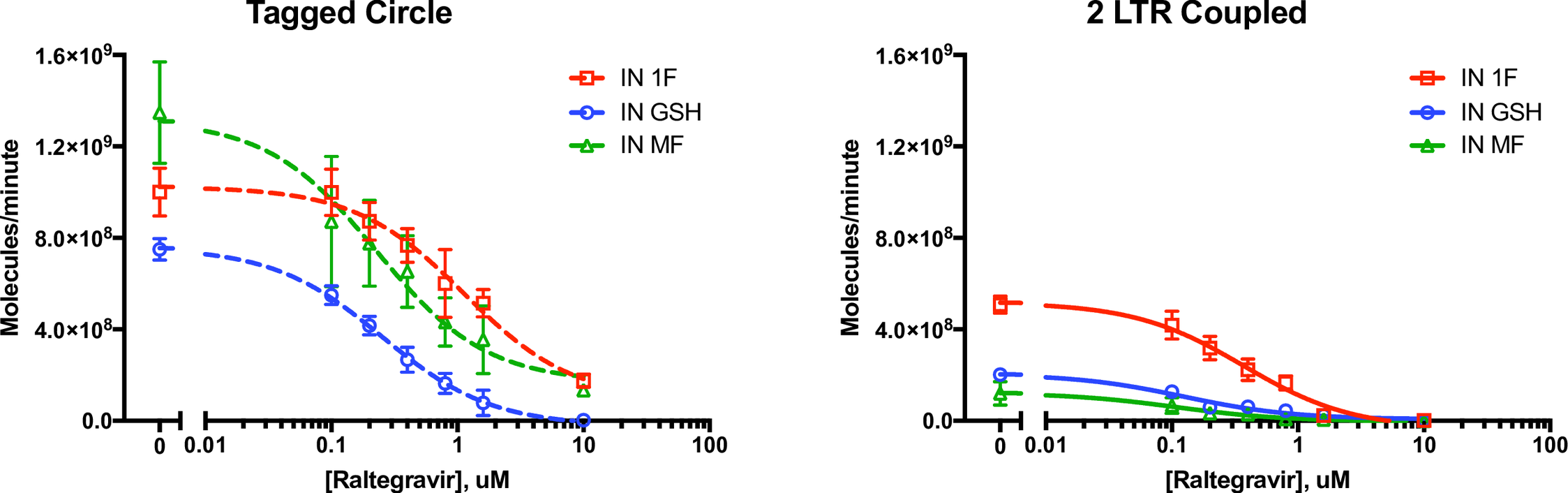

Supplement: Supplementary file 5 — Additional file 5: Figure S5. Effect of raltegravir on strand transfer activity of IN 1F, IN GSH, and IN 1F. Data are the same as in Fig. 3e but plotted as molecules integrated per minute of single strand (left) and concerted integration (right) without normalization. Data plotted as mean ± SD of 3 replicates. [file 12977_2020_537_MOESM5_ESM.tif]

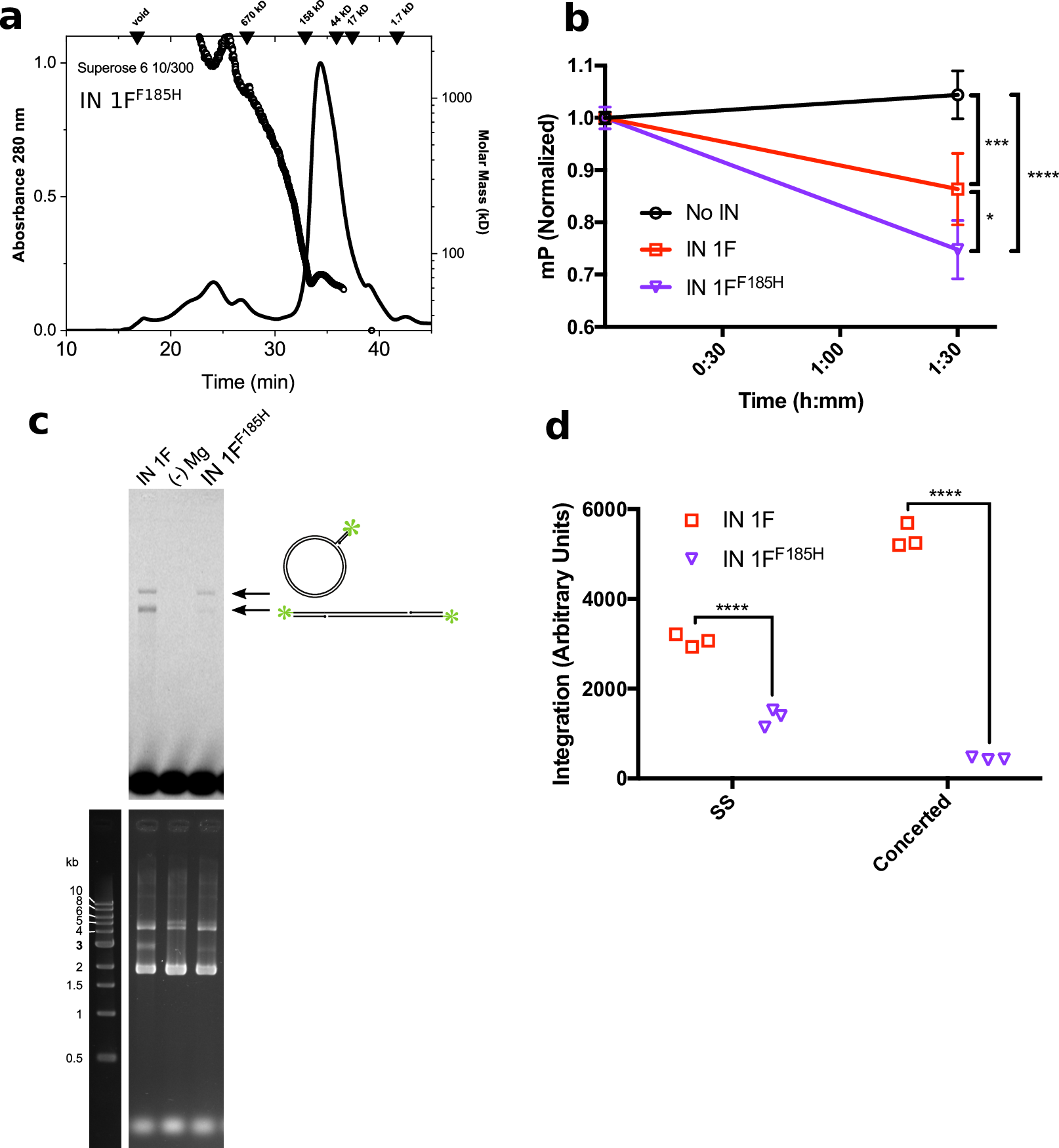

Supplement: Supplementary file 6 — Additional file 6: Figure S6. Biophysical and biochemical characterization of IN 1FF185H. a) SEC-MALS analysis of IN 1FF185H shows a mixture of dimers and higher-order aggregates in solution. b) Quantification of fluorescence polarization assay for 3’ processing. IN 1FF185H performs 3’-processing more rapidly than IN 1F, a difference that reaches statistical significance, but has unclear biological relevance. c) Example gel image of results from strand transfer assay in the presence of Mg2+. d) Quantification of strand transfer activity. The single strand and concerted strand transfer activity of IN 1FF185H is significantly decreased as compared to wild type IN 1F. Data plotted as mean ± SD of 3 replicates. * denotes P < 0.05, *** denotes P = 0.0005, **** denotes P < 0.0001. [file 12977_2020_537_MOESM6_ESM.tif]
